# Closing the Gap Between Recommendation and Uptake: Provider Communication About Low-Dose Aspirin for Preeclampsia Prevention

**DOI:** 10.26502/jppch.74050232

**Published:** 2026-03-25

**Authors:** Sharla M. Smith, Shea Kempsen, Claire Metcalf, Oluoma Obi, Elizabeth Madrigal, Kionna L. Henderson, Megha Ramaswamy

**Affiliations:** 1 University of Kansas Medical Center, Kansas City, KS, USA; 2 Research Scientist II, Health and Wellness Center, Baylor Scott & White Health and Wellness Center, 4500 Spring Avenue, Dallas, TX 75210, USA; 3 Professor and Chair, Department of Health Systems and Population Health, School of Public Health, University of Washington, Seattle, WA, USA

**Keywords:** Hypertensive disorders, Pregnancy, Women, United States, Maternal, Preeclampsia, Worldwide

## Abstract

**Introduction:**

Hypertensive disorders of pregnancy (HDPs), including preeclampsia, disproportionately contribute to preventable maternal morbidity and mortality. This study examined variations in clinician counseling and management of HDPs, provider communication about low-dose aspirin (LDA) prophylaxis, and barriers and facilitators to patient activation.

**Methods:**

A cross-sectional, mixed-methods study was conducted with perinatal care providers at two Midwest academic medical centers (2021–2022). An online survey was completed by 74 providers, followed by semi-structured interviews with a purposive sample of 13 providers. Survey data were analyzed descriptively using SAS 9.4; interview transcripts were analyzed using deductive thematic analysis with intercoder reliability.

**Results:**

All interviewed providers recommended LDA initiation between 12- and 16-weeks’ gestation. Primary barriers included limited medication adherence (43%) and appointment non-attendance (16.5%). Facilitators included LDA’s low cost, over-the-counter availability, and established safety profile. Themes include initiating conversations about LDA with all patients regardless of risk, emphasizing universal LDA uptake, and recognizing continued preeclampsia risk postpartum.

**Conclusions:**

Providers demonstrated familiarity with HDP guidelines but exhibited inconsistencies in counseling. Reframing “patient compliance” as “patient activation” supports equity-centered shared decision-making. Recommended strategies include establishing universal LDA recommendation protocols, expanding Medicaid coverage to reduce insurance barriers, and implementing community-focused public health education initiatives.

## 1. Introduction

Hypertensive disorders of pregnancy (HDPs), including preeclampsia, gestational hypertension, eclampsia, and HELLP syndrome, are a leading cause of preventable maternal morbidity and mortality in the United States [1-3], defined by systolic blood pressure ≥140 mmHg and/or diastolic blood pressure ≥90 mmHg on two occasions at least four hours apart [4]. Preeclampsia affects 2-8% of pregnancies and accounts for more than 70,000 annual maternal deaths worldwide [5], with approximately 60% considered preventable [6]. HDP-related mortality has severe racial and ethnic disparities, with a higher proportion of pregnancy-related deaths attributed among Black (8.2%) and Hispanic (9.7%) women compared to White women (6.7%) in the United States [7,8] Systemic bias [8], inadequate counseling [9], and low uptake of preventative therapies [10] impede clinical and holistic improvements.

Low-dose aspiring (81 mg/day) reduces preeclampsia risk by 10-24% and is recommended by the U.S. Preventive Services Task Force (USPSTF), American College of Obstetricians and Gynecologists (ACOG), and Society for Maternal-Fetal Medicine (SMFM) for initiation between 12- and 16-weeks’ gestation in high-risk individuals [11]. Despite these recommendations, preeclampsia incidence has changed little since guideline adoption [3,12]. Provider counseling practices, including how clinicians communicate risk, prescribe LDA, and navigate patient-level barriers, represent a critical and under-researched area. This study characterizes provider counseling and management of HDPs, implementation of evidence-based LDA prophylaxis, and provider-identified barriers and facilitators to guideline adherence.

## 2. Methods

This cross-sectional, mixed-methods study combined an online survey and semi-structured interviews with perinatal care providers at two Midwest academic medical centers (2021–2022). The study was approved by the Institutional Review Board of the University of Kansas Medical Center; written informed consent was obtained from all participants, who received gift card incentives.

The survey was distributed electronically to attending physicians, residents, fellows, and midwives (n=74). The instrument addressed six domains: demographics, education and awareness, monitoring and recommendations, LDA use, barriers and facilitators, and postpartum follow-up. Data was analyzed using SAS 9.4; categorical variables are reported as frequencies and proportions (Table 1).

From survey respondents, a purposive sample of 13 providers participated in virtual, 30–60-minute semi-structured interviews (4 family medicine physicians, 8 OB/GYN physicians, 1 nurse-midwife). Interview guides addressed HDP counseling, LDA prescribing, barriers to uptake, and postpartum preeclampsia management. Questions were tailored to each participant’s survey responses. Interviews were recorded and transcribed verbatim. Two reviewers conducted deductive thematic analysis with established intercoder reliability to develop code summaries and overarching themes. The five themes identified include Initiating Conversations, Low-dose Aspirin Prescription, Barriers to LDA uptake, Facilitators to LDA uptake, and Postpartum Preeclampsia Diagnosis (Table 2).

## 3. Results

Survey respondents were primarily White (83.8%), aged 25–34 (60.8%) and held doctoral degrees (98.6%). As shown in Table 3, 75.7% of providers reported discussing high blood pressure with all pregnant patients; 23.0% distributed educational materials. All providers (100%) reported recommending LDA to high-risk patients, with most indicating initiation at 12–16 weeks’ gestation. High-risk patients were extremely or very likely to receive increased monitoring (93.2%). Barriers identified in the survey included structural constraints on appointment attendance, knowledge gaps in LDA dosing, and low patient uptake. Facilitators included LDA’s low cost, over-the-counter accessibility, established safety profile, and widespread public familiarity with aspirin.

Interviews conducted contained 13 participants, including a Nurse Midwife (n=1, 7.7%), OB/GYN Physicians (*n*=8, 61.5%), and Family Medicine Physicians (*n*=4, 30.8%) (Table 2). Five themes were identified in physician interviews: 1) Initiating Conversations, 2) Low-Dose Aspirin (LDA) Prescription, 3) Barriers to LDA Uptake, 4) Facilitators to LDA Uptake, and 5) Postpartum Diagnosis, as shown in Table 4.

Among those initiating early counseling, topics included HDP risk factors, a general overview of hypertension in pregnancy, and probing questions to assess patient-specific risk. While all agree that HDP should be discussed during clinical visits, the majority only initiated in-depth conversations with patients with [13] 1 high- risk factors. One provider noted the absence of a standardized institutional protocol for the initial prenatal visit, leading to nonstructured counsel based on provider subjectivity during the critical early gestational weeks.

Providers (n=2) 15.3% initiated HDP conversations with all patients, regardless of risk factors. This was particularly important to medical providers that served low-income or uninsured populations. Providers (n=5) 38.5% initiated HDP conversations only with patients perceived as high- risk. Patients with high-risk factors, including a previous history of cardiovascular disease, preeclampsia, or Black race, had intentional conversations with providers reviewing warning signs and additional blood pressure monitoring.

All providers (100%, n=13) agreed that LDA is a crucial preventive strategy for HDP and early-onset preeclampsia. While recommendations for when to start LDA use minimally vary, consensus for counsel at the end of the first trimester and into the second trimester was recorded. Guidelines for aspirin dosage follow 81 mg/day, but guidelines occasionally differed to up to 162 mg/day, differing by medical institution and accepted clinical practices.

All providers (100%, n=13) recommended initiating LDA between 12- and 16-weeks’ gestation as directed by most globally accepted guidelines; two suggested initiation as early as 5 weeks. One provider reported advising LDA initiation at 18–20 weeks’ gestation for lower-risk patients, diverging from ACOG and USPSTF guidelines. Gestational timelines were seen as recommendations to follow but varied by risk factor, time with patient, and subjectivity of medical providers.

Limited medication adherence was identified as the largest barrier to LDA uptake (43%, n=34). Many providers felt that while they always discussed LDA importance, the uptake was dependent on patient responsibility and use. Appointment non-attendance reflected structural barriers including transportation, childcare obligations, insurance limitations, and employment demands. Formally prescribing LDA, rather than simply recommending it, may improve adherence by signaling clinical importance. Some suggested pairing LDA with prenatal vitamins to encourage regular use. Limited appointment attendance (16.5%, n=13) was also mentioned as a barrier to LDA prophylaxis. Providers could not prescribe or suggest LDA usage if patients did not attend perinatal appointments regularly, but accepted common barriers, especially for low-income or uninsured patients.

LDA Uptake facilitators include general low cost as well as the availability of the medication in most general shopping stores. LDA is a low barrier to entry medication which promotes its continued use throughout the pregnancy and postpartum periods. Low-dose aspirin can be found at any drug store or general store and can be bought over the counter with no insurance premiums or copays. Providers discussed the general acceptability of LDA as safe with common name recognition.

Twenty-six interview comments addressed postpartum preeclampsia, discussing the growth of incidence rate despite the lack of research on postpartum complications. This may signal a growing need for a comorbidity focus in the postpartum period, enhanced by the lack of continuation of care during this time. 7.7% (n=2) of providers described seeing an increasing number of postpartum preeclampsia patient, noting an increasing number of cases of postpartum preeclampsia requiring hospitalization. 46.2% (n=12) addressed postpartum preeclampsia as a clinical concern. The change from OB/GYN provider to family medicine practitioner post-birth was noted where patients typically “fell through the cracks.”

34.6% (n=9) comments discussed barriers in addressing postpartum preeclampsia, including caring for a newborn, long emergency department wait times, and breastfeeding logistics. Providers also identified the absence of a formalized handoff between obstetric and primary care settings as a critical systems-level gap, limiting continuity of monitoring after delivery.

## 4. Discussion

This mixed-methods study identified meaningful inconsistencies between provider-reported and provider-described hypertensive disorders of pregnancy (HDP) counseling practices, suggesting that knowledge of recommendations does not consistently translate into clinical action. Although all respondents demonstrated awareness of current low-dose aspirin (LDA) guidelines, providers’ descriptions of practice revealed that counseling was often reserved for patients perceived as high risk rather than delivered consistently across eligible populations. Notably, while 75.7% of survey respondents reported discussing high blood pressure with all pregnant patients, interview data indicated that most providers initiated these conversations selectively, creating missed opportunities for early prevention among patients with moderate-risk factors and those facing socioeconomic barriers to care. Given that a substantial proportion of HDP-related maternal deaths are considered preventable, these findings underscore how inconsistent counseling may deepen existing disparities even when an effective, low-cost, and widely available preventive therapy exists. These findings are consistent with prior literature showing that only about half of eligible nulliparous patients receive guideline-concordant clinician counseling about aspirin prophylaxis and with commentaries arguing that miscommunication around LDA supports broader, more universal prevention approaches [14].

This study also found that the barriers most frequently cited by providers were medication adherence and appointment attendance reflecting structural constraints more than individual patient shortcomings. Framing these challenges as failures of “compliance” risks obscuring the roles of transportation instability, insurance gaps, childcare demands, employment constraints, and limited continuity of prenatal care. Reframing the problem as one of patient activation and health system support better aligns with equity-centered maternal care because it shifts responsibility toward shared decision-making, anticipatory counseling, and interventions that make adherence feasible in daily life. In this context, inconsistent clinical action around LDA recommendation or prescription is not merely a communication issue but a systems issue with implications for maternal outcomes. This interpretation is supported by studies documenting racial disparities in aspirin prophylaxis use, broader evidence linking structural racism and implicit bias to maternal health inequities, and patient-facing information gaps that may limit understanding of preeclampsia prevention outside the clinical encounter [15].

Finally, our findings support universal HDP counseling and more standardized LDA prescribing practices as equitable strategies to reduce practice variation and improve preventive care delivery. Universal counseling would establish a minimum standard of care for all pregnant patients, while formal prescribing rather than informal recommendation alone may better communicate the clinical importance and urgency of aspirin use. Because LDA is inexpensive, widely available, and supported by national recommendations for patients at increased risk of preeclampsia, broader implementation may help mitigate inconsistent recognition of moderate-risk factors and reduce the influence of implicit bias in selective counseling. Providers in this study also emphasized the need for structural reforms, including expanded postpartum Medicaid coverage, stronger continuity of care before and after birth, and community-centered public health education regarding aspirin use in pregnancy. Together, these findings suggest that optimizing HDP prevention requires both standardized clinical protocols and structural support that make adherence possible. This conclusion aligns with USPSTF evidence supporting aspirin to prevent preeclampsia-related morbidity, with recent quality-improvement work focused on improving counseling among patients with moderate-risk factors, and with the growing literature calling for system-level reforms to address persistent racial inequities in maternal health [16-41]

## 5. Implications

Future efforts to reduce hypertensive disorders of pregnancy should extend beyond selective risk-based counseling to universal low-dose aspirin prescribing supported by strategies that reduce implicit bias and structural barriers to uptake. Standardized early counseling and prescribing, particularly in the 12- to 16-week window, may improve adherence and reduce inequities in maternal outcomes related to race, income, and insurance status.

## 6. Limitations

While this study has numerous strengths including the mixed-methods design enabling triangulation of findings, and inclusion of providers from multiple specialties and training levels, some limitations exist. Limitations include the small interview sample, limited provider demographic diversity (predominantly White), and geographic concentration at two Midwest academic medical centers. The absence of the patient's perspective is a key limitation; future research should integrate patient-reported experiences to fully characterize barriers and facilitators. Future studies should also examine contributions of midwives, community health workers, and doulas, as well as perspectives of racially diverse providers who may offer insights into culturally congruent care.

## 7. Conclusions

Providers demonstrate general familiarity with LDA guidelines for HDP prevention but exhibit significant variation in counseling practices. Reframing patient compliance as patient activation, standardizing prenatal counseling protocols, and expanding postpartum support structures are actionable strategies to improve HDP prevention and management. Policy changes, including universal prescription of low-dose aspirin, could promote equitable changes in maternal health outcomes and alleviate HDP-related health disparities.

## Figures and Tables

**Table 1:** Survey Respondent Demographics (N=74).

Characteristic	N (%)
**Race/Ethnicity**	
White	62 (83.8%)
Hispanic or Latino	4 (5.4%)
Native American/American Indian or Asian/Pacific Islander	4 (5.4%)
Other	2 (2.7%)
**Age Range**	
25–34	45 (60.8%)
35–44	21 (28.4%)
45–54	7 (9.5%)
55+	1 (1.4%)
**Education**	
Doctorate	73 (98.6%)
Master’s degree	1 (1.4%)

**Table 2:** Interview Participant Demographics (N=13).

Characteristic	N (%)
**Race/Ethnicity**	
White	10 (76.9%)
Hispanic or Latino	1 (7.7%)
Native American/American Indian or Asian/Pacific Islander	1 (7.7%)
Other (White/Asian)	1 (7.7%)
**Age Range**	
25–34	4 (30.8%)
35–44	6 (46.2%)
45–54	2 (15.4%)
55+	1 (7.7%)
**Specialty**	
OB/GYN Physician	8 (61.5%)
Family Medicine Physician	4 (30.8%)
Nurse-Midwife	1 (7.7%)
**Education**	
Doctorate	13 (100.0%)

**Table 3:** Survey Results (N=74).

Category & Questions	Responses N (%)
**Education & Awareness** Do you discuss high blood pressure/hypertension with all pregnant women? What does the discussion about high blood pressure cover? (Check all that apply) Do you provide educational materials about high blood pressure/hypertension to all pregnant women?	Yes 50 (75.7%) No 17 (22.9%) N/A 7 (9.6%) Risk factor education 44 (88.0%) Healthy pregnancy 43 (86.0%) Chronic Hypertension 36 (72.0%) Weight 31 (62.0%) Diabetes 27 (54.0%) Family history 26 (52.0%) Not Answered 17 (25.4%) Yes 15 (23.0%) No 52 (70.2%) N/A 7 (9.5%)
**Monitoring & Recommendations** If risk factors are present for hypertension and/or EOP, how likely are you to provide additional monitoring for the patient? What additional recommendations do you give for patients at risk for EOP? (Check all that apply)	Not at all likely 1 (1.3%) Somewhat likely 21 (28.4%) Extremely likely 52 (70.1%) LDA Prescription 74 (100%) Educate patient 73 (98.6%) Analysis of vitals & lab work 59 (79.7%) Discuss lifestyle modifications 58 (78.4%) Increased ultrasound & BP 51 (68.9%) Daily self BP checks 43 (58.1%) Consultation with patient 45 (60.8%) More frequent physician visits 42 (56.8%) Referral to high-risk physician 40 (54.1%) Risk factor checklist 38 (51.4%) Other 7 (9.4%)
**Low-Dose Aspirin** How likely are you to prescribe aspirin to patients at risk for preeclampsia? Would you prescribe aspirin if a patient has at least one risk factor? At what point would you initiate low-dose aspirin for a pregnant woman at risk for hypertension/EOP? Would you recommend a duration of low-dose aspirin treatment until delivery?	Not at all likely 0 (0.0%) Somewhat likely 8 (10.8%) Extremely likely 66 (89.2%) Yes 52 (70.2%) No 22 (29.8%) First trimester, weeks 1-4 9 (12.2%) First trimester, 5-8 weeks 6 (8.1%) First trimester, 9-13 weeks 49 (66.2%) Second trimester, 14-17 weeks 34 (46.0%) Second trimester, 18-22 weeks 16 (21.6%) Second trimester, 23-27 weeks 16 (21.6%) Third trimester, 28-31 weeks 6 (8.1%) Third trimester, 32-35 weeks 6 (8.1%) Third trimester, 36-40 weeks 4 (5.4%) Missing 9 (12.2%) Yes 54 (73.0%) No 13 (17.6%) N/A 7 (9.4%)
**Barriers & Facilitators to Implementing Clinical Guidelines** Barriers Limited patient compliance Unaware of appropriate recommendation Limited medication adherence Lack of shared decision-making Limited evidence around prevention of EOP Other Facilitators Cost of low-dose aspirin Evidence of limited harm Evidence of effectiveness Increased access USPSTF recommendations Other	40 (54.1%) 40 (54.1%) 38 (51.4%) 23 (31.1%) 10 (13.5%) 7 (9.5%) 64 (86.5%) 62 (83.8%) 61 (82.4%) 49 (66.2%) 49 (66.2%) 5 (6.8%)
**Postpartum Preeclampsia** Postpartum Recommendations for Birthing People who Experienced Preeclampsia, Eclampsia, Hypertension, or HELLP Syndrome Referral to primary care physician More frequent postpartum visits Educational material on symptoms and causes of postpartum preeclampsia Follow-up appointment to discuss what the patient experienced during delivery Multiple interventions/screenings Other	51 (68.9%) 51 (68.9%) 50 (67.6%) 47 (63.5%) 17 (23.0%) 4 (5.4%)

**Table 4:** Interview Results (N=13).

Theme	Subtheme	Quote
Initiating Conversations		
	All Patient Counselling	*“Hypertension is so prevalent that it is part of what I discuss with basically all my patients… we know that that is something that can lead to pretty significant morbidity and even mortality…”*
	High-Risk Patient Counselling	*“I don’t routinely talk about the risks of high blood pressure or preeclampsia with all patients. I typically will only talk about it with people who have a little bit more of an increased risk…”*
Low-Dose Aspirin Prescription		
	Gestational Timeline of Aspirin Prescription	*“You should start it roughly in the late first trimester. So that's typically just when I start it. I know it’s supposed to be before 12-16 weeks so, yeah, it's usually roughly around the end of the first trimester that I'll start it or have a conversation with them about starting it”.*
Barriers to LDA Uptake		
	Medication Adherence	*“…How busy your life is… you’re maybe chasing kids and, ‘Oh, forgot to take my aspirin,’ it’s not a priority…”*
	Appointment Adherence	*“I think, one, like I mentioned, is getting patients in for prenatal care early, I like that that is a marker that, of health that everyone is trying to do nationally, is get people in early for their visits, so a lot of education around that, but it's also a larger system issue, like it's just hard for patients to get in sometimes”.*
Facilitators to LDA Uptake		
	Low Cost	*“Another thing is like socioeconomically, you know, it's a very inexpensive medication to purchase for our patients that don't have insurance”.*
	Availability and Access	*“Most of our patients just prefer to go pick it up at Costco—you get a barrel of it for $5—so I just ask each visit, are you, how is your aspirin going…”*
Postpartum Preeclampsia Diagnosis		
	Increased postpartum preeclampsia	*“I have started noticing that we have more patients it seems that need postpartum admission for symptomatic blood pressure after their delivery consistent with the diagnosis of postpartum preeclampsia.”*
	Postpartum Preeclampsia Patient Challenges	*“I find that one of the most challenging things is, as a postpartum patient it's different, you now have a small infant to be taking care of, and so it's really challenging for patients to seek medical attention like, you know, go to the ER if your blood pressure's high, like this is not something that people want to do, like the ER wait times are so, so, so long. If you have an infant you're breastfeeding, that really does not make sense.”*
